# Deficiency of the ribosomal protein uS10 (RPS20) reorganizes human cells translatome according to the abundance, CDS length and GC content of mRNAs

**DOI:** 10.1098/rsob.230366

**Published:** 2024-01-31

**Authors:** Yueming Tian, Elena S. Babaylova, Alexander V. Gopanenko, Alexey E. Tupikin, Marsel R. Kabilov, Alexey A. Malygin

**Affiliations:** Institute of Chemical Biology and Fundamental Medicine, Siberian Branch of the Russian Academy of Sciences, Novosibirsk 630090, Russia

**Keywords:** ribosomal protein uS10, ribosomal deficiency, RNA-seq, mRNA, translation regulation

## Abstract

Ribosomal protein uS10, a product of the *RPS20* gene, is an essential constituent of the small (40S) subunit of the human ribosome. Disruptive mutations in its gene are associated with a predisposition to hereditary colorectal carcinoma. Here, using HEK293T cells, we show that a deficiency of this protein leads to a decrease in the level of ribosomes (ribosomal shortage). RNA sequencing of the total and polysome-associated mRNA samples reveals hundreds of genes differentially expressed in the transcriptome (t)DEGs and translatome (p)DEGs under conditions of uS10 deficiency. We demonstrate that the (t)DEG and (p)DEG sets partially overlap, determine genes with altered translational efficiency (TE) and identify cellular processes affected by uS10 deficiency-induced ribosomal shortage. We reveal that translated mRNAs of upregulated (p)DEGs and genes with altered TE in uS10-deficient cells are generally more abundant and that their GC contents are significantly lower than those of the respective downregulated sets. We also observed that upregulated (p)DEGs have longer coding sequences. Based on our findings, we propose a combinatorial model describing the process of reorganization of mRNA translation under conditions of ribosomal shortage. Our results reveal rules according to which ribosomal shortage reorganizes the transcriptome and translatome repertoires of actively proliferating cells.

## Graphical abstract

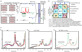

## 1. Introduction

Ribosomal protein uS10 (RPS20) is an indispensable constituent of the human ribosome. It is located in the head of the 40S ribosomal subunit and, in addition to 18S rRNA, contacts ribosomal proteins uS3 and uS14, extending one of its regions towards the mRNA-binding channel, although not reaching it [1]. During the assembly of the head of the 40S subunit, which is formed around the 3′-terminal domain of 18S rRNA, these ribosomal proteins within the corresponding cluster with the eS10 protein settle into their proper sites after the release of assembly factors ENP1 and LTV1 from the beak of the subunit head in the middle stage of 40S subunit assembly [2]. Ribosomal proteins uS3, uS10 and uS14 belong to the group of proteins whose depletion in HeLa cells practically does not affect the processing of the 5′-ETS region of 45S pre-rRNA but is characterized by defects in the processing of the ITS1 region during 18S rRNA maturation [3,4]. As a result, the level of mature 40S ribosomal subunits decreases in cells upon uS10 knockdown. As a component of the ribosome, uS10 is involved in the ribosome-associated quality control (RQC) pathway (e.g. [5]), which starts with the recognition of prematurely stalled translating ribosomes and their ubiquitination of specific ribosomal proteins and is eventually completed by degradation of the aberrant mRNAs and synthesized peptides. In mammalian cells, the ubiquitination of uS10 (together with eS10 and uS3 ribosomal proteins) at the K4/8 residues in abnormally stalled ribosomes is the first step of RQC [6].

Model organisms with a knockout of the uS10 ribosomal protein are unknown, apparently due to the critical need for this protein for cell viability. In humans, hereditary defects in ribosome biogenesis or protein synthesis caused by the loss of function of one allele of a ribosomal protein-encoding gene lead to various abnormalities called ribosomopathies [7] and are regarded as a serious risk factor for cancer development [8]. Analysis of the genomic information from several families with a hereditary predisposition to non-polyposis colorectal cancer (CRC) revealed various mutations in the coding region of the RPS20 gene encoding the uS10 ribosomal protein. Among them were disruptive mutations such as an insertion and a deletion, causing a frameshift and synthesis of truncated protein forms (p.V50SfsX23 and p.L61EfsX11) [9], and missense mutations leading to the substitution of amino acid residues (p.V54L and p.E33V) [9,10]. Meanwhile, the reasons why uS10 haploinsufficiency can lead to the development of CRC remain unknown.

We have recently shown how mutant forms of the uS10 protein, resulting from the expression by HEK293T cells of genetic constructs containing copies of the gene encoding uS10 with mutations authentic to those found in patients with a predisposition to CRC, change the cellular transcriptome [11]. Using RNA-seq and subsequent bioinformatics analysis, we found that among the limited number of genes expressed differentially in the cells generating mutant uS10 forms, there were upregulated genes directly associated with the progression of CRC (e.g. *PPM1D* and *PIGN*). An RNA-seq-based approach to determining changes in global gene expression at the transcriptome and translatome levels upon ribosomal protein deficiency has also proven to be productive in studying the consequences of deficiency of various ribosomal proteins [12]. Thus, when analysing the changes that occur in the cell transcriptomes upon knockdown of non-essential ribosomal proteins eL29 or eL38, hundreds of genes were identified, the expression of which increased or, conversely, decreased, and it was found that the patterns of the changed genes were different [13,14]. Comparison of changes in the sets of total cellular mRNAs and mRNAs translated in cells upon knockdown of the obligatory 60S subunit ribosomal protein uL5 showed differences between transcriptionally regulated genes and genes with altered translational efficiency [15]. Thus, it was of interest to determine how a decrease in the level of the obligatory 40S subunit ribosomal protein uS10 changes the composition of the cell transcriptome and the set of translated mRNAs.

In this work, with the use of the RNA interference approach to knockdown the uS10 protein in HEK293T cells, we reveal a significant decrease in the level of 40S ribosomal subunits, 80S monosomes and polysomes in uS10-knockdown cells compared to control ones, which allows us to suppose ribosomal shortage in the uS10-deficient cells. By applying RNA-seq to the total cellular mRNA and to the set of mRNAs translated in polysomes, we determined genes expressed differentially depending on the uS10 level and showed dissimilarities in changes in the compositions of total and translated mRNAs. Thus, we found an increase in the total level of mRNAs of many genes, including several transcription factors and oncogenes, and a decrease in the total level of mRNAs of some specific gene families; however, this decrease was not accompanied by a similar decrease in the translation of these mRNAs. By analysing up- and downregulated genes, we conclude that many genes lack coordination in the regulation of expression between transcription and translation levels. We show that changes in the efficiency of mRNA translation in uS10-deficient cells are correlated with mRNA abundance, coding sequence (CDS) length and GC content, which may be described by a model of combinatorial selection of mRNAs upon ribosomal shortage.

## 2. Results

### 2.1. Knockdown of ribosomal protein uS10 in HEK293T cells

**Figure 1.**
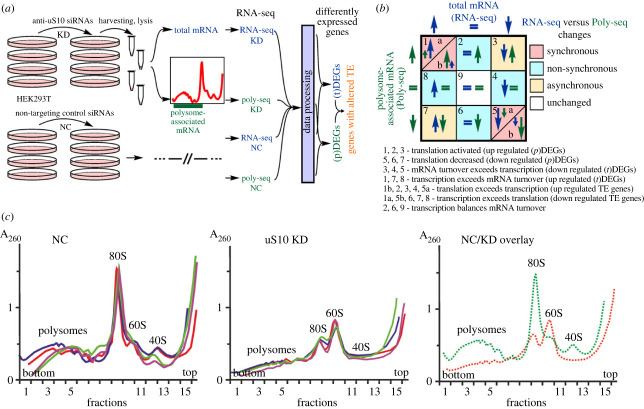
Knockdown of the ribosomal protein uS10 in HEK293T cells and analysis of changes in the cellular transcriptome and translatome associated with uS10 deficiency. (*a*) The workflow of the experiment. Cells were transfected with siRNAs (KD, targeted against uS10 mRNA or NC, a control non-targeting siRNAs) and, after cultivation, mRNA samples were obtained either from the total cytoplasmic lysate or from polysome-containing fractions collected after centrifugation of the lysate in a sucrose gradient; RNA was sequenced, and differentially expressed genes (DEGs) were determined. (*b*) Principal scheme illustrating possible variants of overlapping differences in gene expression in the transcriptome and translatome and their interpretation. (*c*) Polysome profiles obtained by sucrose density gradient centrifugation of the lysates of cells transfected with uS10 mRNA-specific siRNAs (uS10 KD) and non-targeting control siRNAs (NC). Each figure shows an overlay of polysome profiles for four replicates of the experiment. Panel ‘NC/KD overlay’, a superposition of the averaged polysome profiles over four replicates obtained with uS10-deficient cells (red line) and cells treated with non-targeting siRNAs (green line). The peaks of 60S and 40S ribosomal subunits, 80S monosomes and polysomes are designated.

To reduce the level of ribosomal protein uS10 in cells, we applied the RNA interference approach with the use of specific siRNAs targeted to two regions in the CDS of uS10 mRNA. The experiment was carried out as follows (figure 1*a,b*). HEK293T cells were transfected with either specific or control non-targeting siRNAs in four biological replicates and harvested at 36 h. An RT-qPCR analysis of the uS10 mRNA level in the treated cells showed that it decreased by approximately ninefold after transfection with specific siRNAs compared to the control cells (electronic supplementary material, figure S1). We deliberately avoided measuring the change in the level of uS10 here and limited ourselves to measuring the level of its mRNA, since essential ribosomal proteins are present in the cell mostly as parts of ribosomal subunits, and a change in their content can be concluded from a change in the content of the subunits. The harvested cells were lysed, a portion of the lysate was centrifuged in a sucrose gradient, and RNA was isolated both from the total lysate and from the polysomal fractions of the sucrose gradient for subsequent RNA-seq (figure 1*a*). To distinguish between the data obtained for total and polysome-associated mRNAs, we named them RNA-seq and Poly-seq, respectively. Polysome profiles obtained by the centrifugation of the lysates of uS10-knockdown cells and control cells in the sucrose gradients differed drastically. In cells treated with specific siRNAs, the levels of polysomes, 80S monosomes and 40S ribosomal subunits were significantly reduced (the decrease in the level of 80S monosomes was approximately threefold), while the level of 60S ribosomal subunits was markedly increased (figure 1*c*). Analysing the content of individual ribosomal proteins in fractions of ribosomes and polysomes using Western blotting, we did not find a large disproportion in the content of these proteins, however, this method is rather crude and cannot provide a complete guarantee of the stoichiometry of ribosomal proteins in subunits (electronic supplementary material, figure S2). All of the above suggests that a decrease in the uS10 mRNA level may cause insufficient synthesis of uS10 and its deficiency in cells, which, given the indispensability of this protein for the assembly of 40S subunits, leads to a shortage of ribosomes in general.

### 2.2. Analysis of RNA-Seq data obtained with total and polysome-associated mRNAs and differentially expressed genes between cells with and without uS10 knockdown

To determine differences in the content of total and polysome-associated mRNAs between uS10-knockdown and control cells, RNA-seq for the respective samples of isolated RNA was carried out. The evaluation of the quality of the sequencing data by principal component analysis (PCA) showed a high degree of clustering between four biological replicates in both RNA-seq and Poly-seq sequencing modes (electronic supplementary material, figure S3), implying that the data obtained were suitable for further processing. When mapped to the reference human genome, the filtered and quality-checked raw sequencing reads fell mainly into the exonic regions of the protein-coding genes (electronic supplementary material, table S3). In total, over 12 000 genes were covered by sequencing reads in both RNA-seq and Poly-seq modes (electronic supplementary material, tables S4 and S5).

**Figure 2.**
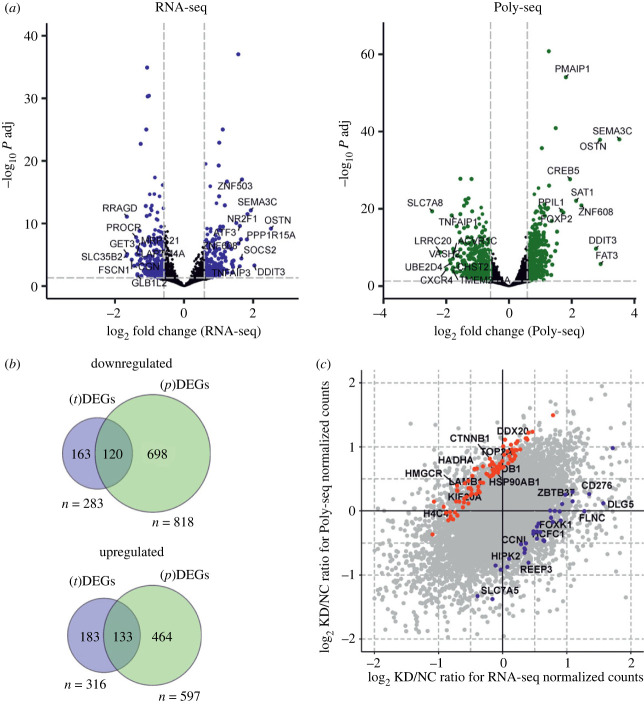
Characterization of DEGs under uS10 deficiency in HEK293T cells. (*a*) Volcano plots representing the DEG analysis results of total (RNA-seq) and polysome-associated (Poly-seq) mRNA sequencing data. Dashed lines show the cutoff parameters of fold change and adjusted p. Coloured dots correspond to genes with statistically significant changes in expression levels. (*b*) Venn diagrams showing the overlap of downregulated and upregulated (t)DEGs and (p)DEGs. (*с*) Scatter plot showing the ratio of the log2-transformed ratios of means of normalized reads between non-targeting control (NC) and uS10 knockdown (KD) samples for RNA-seq and Poly-seq data. Red and blue dots correspond to genes with altered TE (translation efficiency, *p* adj < 0.05, |LFC| > 0.322) with increased and decreased translational efficiencies, respectively. Several of the top upregulated and downregulated genes with altered TE (according to LFC values) are designated with gene names.

Differential expression analysis performed with the sequencing data obtained with the total cellular or only polysome-associated mRNA isolated from cells with or without knockdown of uS10 revealed sets of genes whose expression was altered at the transcriptional and translational levels (figure 1*b*). From these sets, the genes whose mRNA contents were statistically significantly altered in the samples of total mRNA (abbreviated as (t)DEGs) or polysome-associated mRNA, (p)DEGs, were determined using the defined cutoff parameters. Specifically, the adjusted *p*-value (*p* adj) was taken below 0.05, and the minimal absolute shrunken Log2 Fold Change value (LFC_Shrunken) was limited to 0.585 to only consider the genes with more than a 1.5-fold change in the expression level (figure 2*a*). We identified a set of tDEGs consisting of 283 downregulated and 316 upregulated genes (electronic supplementary material, table S4), as well as a set of pDEGs comprising 818 and 597 such genes (electronic supplementary material, table S5), respectively. Notably, the overlaps between the sets of (t)DEGs and (p)DEGs in both down- and upregulated gene groups were unexpectedly small, 120 and 133, respectively (figure 2*b*), meaning that the differential expression of genes at the level of translation was not completely derived from that at the level of transcription. This suggests that regulation of gene expression in uS10 knockdown cells occurs both transcriptionally and translationally.

To validate the results of differential gene expression analyses performed with the RNA-seq and Poly-seq data, we carried out RT-qPCR analysis for a representative group of DEGs. The values of changes in the expression of these genes at the transcription and translation levels, estimated by RT-qPCR, correlated well with the respective values obtained using the above analysis using the NGS data (electronic supplementary material, figure S4).

### 2.3. Cellular processes associated with up- and downregulated DEGs and genes with altered translational efficiency

**Figure 3.**
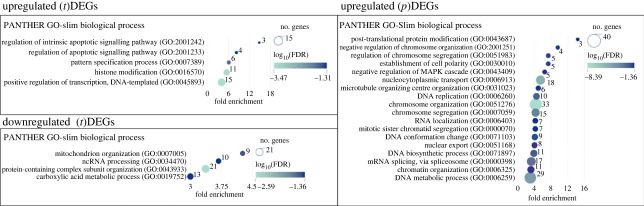
Top biological process terms in Gene Ontology (GO) enrichment analysis (PANTHER GO-slim annotation) performed for (t)DEG and upregulated (p)DEG sets. The most specific GO subclasses in their own hierarchies with an enrichment factor of more than 3 are presented in panels. The legends for the colour (fold discovery rate, FDR) and size (number of genes) of dots are shown on the right.

Since the set of ribosome-associated mRNAs is an integral part of the total cellular mRNAs, one would expect (p)DEGs to correlate with (t)DEGs, and vice versa. The up- and downregulated (t)DEG sets partially overlapped with the corresponding (p)DEG sets (figure 2*b*). This suggests that changes in the expression of individual genes at the transcription level are accompanied by similar changes at the translation level, and vice versa; changes in gene expression at the transcription level affect the overall mRNA level of these genes. However, moderate matching of (t)DEGs and (p)DEGs may indicate that translational regulation of some genes is largely independent of transcriptional regulation. In addition, the sets of up- and downregulated (p)DEG are significantly larger than the corresponding sets of (t)DEG, which may mean that the regulation of gene expression occurs mostly at the level of translation. Gene ontology enrichment (GO) analysis for these up- and downregulated (t)DEG and (p)DEG sets also revealed significant differences between them (figure 3). A set of genes from a group of 316 activated (t)DEGs showed enrichment in a small number of GO biological process terms, which included processes related to histone modification, positive regulation of transcription and several others. It is noteworthy that among the activated genes, there are genes encoding various transcription factors and oncogenes (*TP53*, *MDM2*, *JUN*, *GADD45A* and others), which could change the overall mRNA repertoire of cells.

The number of GO biological process terms enriched in genes from the group of downregulated (t)DEGs of 283 genes was also small and included processes associated with mitochondrial organization and protein-containing complex subunit organization (containing many ribosomal protein genes). Notably, among downregulated (t)DEGs, there was a proto-oncogenic gene *MYC,* known as one of the key regulators of ribosome biogenesis [16] with many activities that stimulate the biogenesis of ribosome constituents, including the activation of genes encoding ribosomal proteins [17]. Therefore, one can assume that a decrease in the overall level of mRNAs of ribosomal proteins was caused, at least in part, by a decrease in the activity of MYC. It should also be noted that for the downregulated (p)DEG set (the largest group with 818 members), no gene enrichment was observed in any GO term. This means that a decrease in transcription of some particular genes caused by a deficiency of the ribosomal protein uS10 is not accompanied by a similar decrease in the translation of their mRNAs. Hence, the translational efficiency (TE) of these genes should increase. Indeed, analysis of GO enrichment for 597 genes present in the upregulated (p)DEGs revealed many specific processes in which these genes are involved (figure 3). These genes were mainly associated with the processes of chromatin organization, DNA metabolism, splicing and protein transport. The significantly greater number of the GO terms gene enrichment for upregulated (p)DEGs than for (t)DEGs may indicate that, during ribosomal shortage, gene expression is regulated more translationally than at the level of transcription. In addition, the absence of GO gene enrichment for downregulated (p)DEGs with a large gene enrichment for upregulated (p)DEGs may indicate that such regulation occurs mainly in the form of activation of translation of specific mRNAs, whereas downregulation of mRNA translation occurs passively.

Given that the TE of a gene is defined as the ratio of its expression at the level of the translatome to that at the level of the transcriptome, it can be noted that the TE of a gene characterizes the absolute difference between these two levels of expression, regardless of how the expression changes. Using the cutoff parameters of statistical significance, we defined genes with altered TE both up- and downregulated ones (figure 2*c*; electronic supplementary material, table S6) and analysed their enrichment in GO terms.

Among the 239 genes with upregulated TE, there were genes mostly enriched in the processes of ribonucleoprotein complex metabolism, cytoplasmic translation, telomere organization and several others (electronic supplementary material, figure S5). No significant GO process enrichment was found for 71 genes with downregulated TE, similar to downregulated (p)DEGs. Thus, along with obvious changes in gene expression at the transcriptional level, uS10 knockdown also leads to an increase in the expression of many genes at the level of translation, which are independent of transcriptional events (i.e. these genes lack coordination in the regulation of expression at these two levels).

### 2.4. The pathway of translatome reorganization in uS10-deficient cells

**Figure 4.**
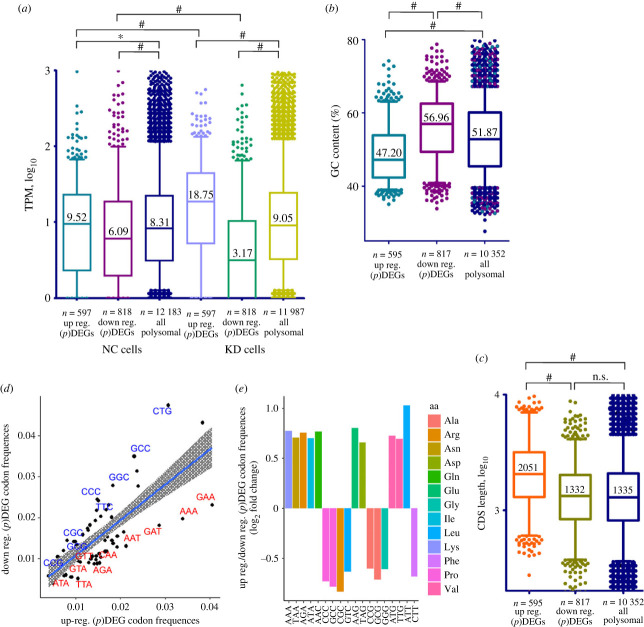
Comparative analyses of the mRNA features for upregulated and downregulated (p)DEGs. (*a*) Distribution of the relative mRNA abundances presented as TPM (transcripts per million) values of the respective genes (by four biological replicates) for up- and downregulated (p)DEGs and for genes corresponding to all sequenced polysomal mRNAs. Box and whiskers (5–95 percentile) plots for the mRNAs from cells treated with non-targeting siRNAs (NC cells) and uS10 knockdown cells (KD cells) are presented. (*b*) Box and whiskers plot for the GC content in the CDS of mRNAs corresponding to up- and downregulated (p)DEGs and to all sequenced polysome-associated mRNAs. (*c*) The same for the CDS length. The median values are designated; ^#^*p* < 0.0001, *p < 0.05 (Mann–Whitney test); n.s., not significant. (*d*) Scatter plot showing the codon frequencies in mRNAs for the upregulated and downregulated (p)DEG sets. (*e*) The most representative changes in the frequencies of codons in mRNAs for upregulated and downregulated (p)DEGs.

To reveal a possible rationale for the activation of translation of specific mRNAs, we tried to find differences in mRNA structural features between up- and downregulated (p)DEGs and genes with altered TE. We noted that the sequencing coverage (a baseMean value obtained in an RNA-seq experiment) of mRNAs of upregulated (p)DEGs was generally much greater than that of mRNAs of downregulated ones, indicating that the cellular representation of the mRNAs of upregulated (p)DEGs was significantly higher than that of downregulated (p)DEGs. By calculating and analysing the TPM values (transcripts per million) as the mRNA abundance measure, we found that the difference between median TPMs for up- and downregulated (p)DEGs increased significantly in uS10-deficient cells compared to control cells (figure 4*a*; electronic supplementary material, tables S5 and S6). Thus, during the ribosomal shortage, more abundant mRNAs gain an advantage in translation compared to less represented mRNAs. This observation may explain to some extent the fact that we found many ribosomal protein genes among translationally activated genes, even if they were downregulated at the transcription level, because ribosomal protein mRNAs are among the most abundant cellular mRNAs [18].

It is noteworthy that the CDS length and CDS GC content of the mRNAs of up- and downregulated (p)DEGs were also different (figure 4*b,c*; electronic supplementary material, tables S5 and S6). The median CDS length for mRNAs of upregulated (p)DEGs was significantly higher than that of downregulated ones or that of sequenced polysomal mRNAs. By contrast, the median level of the GC content of CDS for mRNAs of upregulated genes was much lower than that for the entire set of cellular mRNAs, whereas this parameter for mRNAs of downregulated genes was higher (figure 4*b*; electronic supplementary material, tables S5 and S6). Correspondingly, the codon frequencies for mRNAs of (p)DEGs with increased and decreased expression also changed. Among the former, there were mRNAs with an increased frequency of AT-rich codons, and among the latter, there was a higher frequency of mRNAs with GC-rich codons (figure 4*d,e*). Since the increased GC content correlates with the melting temperature of the mRNA structure, we proposed that mRNAs with a weaker secondary structure are more preferably translated under conditions of ribosomal shortage than highly structured mRNAs.

**Figure 5.**
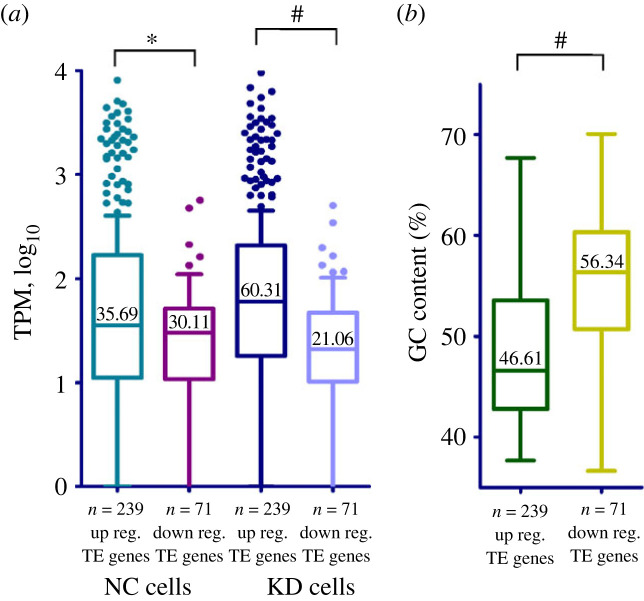
Comparative analyses of the mRNA features for genes with upregulated and downregulated TE. (*a*) Distribution of the relative mRNA abundances (as TPM) for genes with altered TE in NC and KD cells. (*b*) The GC content in CDS of mRNAs corresponding to genes with up- and downregulated TE. Designations as in figure 4.

Similar differences were also observed between the sets of genes with up- and downregulated TE. Like with (p)DEGs, the TPM range between up- and downregulated TE genes was significantly increased in KD cells compared to control cells, and the GC content of mRNAs of genes with upregulated TE was drastically lower than that of genes with downregulated TE (figure 5*a,b*; electronic supplementary material, table S6). Accordingly, the frequencies of AT-rich and GC-rich codons in genes with up- and downregulated TE were very similar to those of (p)DEGs (electronic supplementary material, figure S6). However, the median CDS length of genes with downregulated TE (1905) turned out to be only slightly less than that of genes with upregulated TE (2163), which is most probably due to the small number of identified genes with downregulated TE, when other selection factors considered above play a significant role.

**Figure 6.**
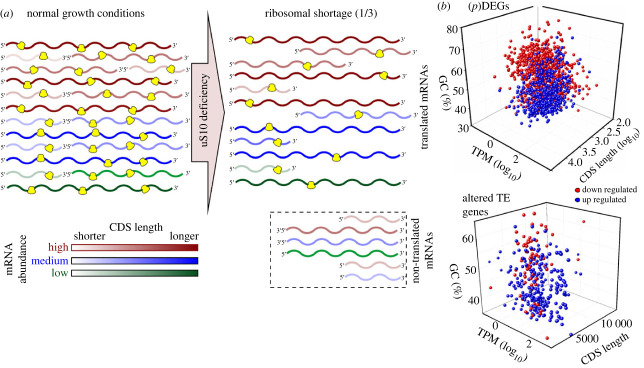
Reorganization of the translatome of human cells under conditions of ribosomal shortage. (*a*) Scheme depicting the proposed mechanism of changes in the content of translated mRNAs during ribosomal shortage caused by a deficiency of the ribosomal protein uS10. When the number of ribosomes is largely reduced (for example, to about one-third), their density per mRNA decreases and a significant portion of mRNAs becomes unoccupied by ribosomes. mRNAs with shorter CDSs and low abundances are more likely to become free of ribosomes, so the relative levels of polysome-associated mRNAs increase for abundant mRNAs with longer CDSs. (*b*) Scatter plots for the mRNAs of upregulated (blue) and downregulated (red) (p)DEGs and genes with altered TE distribution in uS10-deficient cells according to their abundance (TPM), GC content and CDS length.

Thus, we can propose the following scheme of changes in the translatome of cells with knockdown of uS10. Since this protein is obligatory for the maturation of 40S ribosomal subunits, its deficiency reduces the production of mature 40S subunits necessary for the formation of ribosomes and causes a lack of ribosomes in the cell, which could be defined as a ribosomal shortage. Although the overall level of translation and the density of ribosomes per mRNA decrease under these conditions, the most abundantly represented mRNAs still remain involved in translation, while the least represented mRNAs may lose ribosomes (figure 6*a*). Under this scheme, the translatome should also be enriched in mRNAs with longer CDSs, since such mRNAs remain bound to ribosomes for a longer time. The GC content may affect the translation of mRNA because GC-rich mRNA sequences tend to have a complex secondary structure that makes it difficult for ribosomes to move, although a lower abundance of tRNAs that recognize GC-rich codons cannot be ruled out.

Thus, with a deficiency of ribosomal protein uS10, gene expression is regulated both transcriptionally and translationally, and an additive effect leads to a change in the cellular mRNA landscape. On the one hand, changes in the transcription of the genes of several transcription factors alter the production of many mRNAs. On the other hand, the effect of ribosomal shortage caused by a ribosomal protein deficiency reduces the availability of ribosomes for certain categories of mRNAs, depending on the length and GC content of their CDSs and the level in the cell (figure 6*b*).

## 3. Discussion

To track changes in the content of the cell transcriptome and translatome caused by a deficiency of a single ribosomal protein, we induced knockdown of the ribosomal protein uS10 in HEK293T cells and, by applying RNA-seq to total mRNA and translated mRNAs, studied the patterns of differentially expressed genes. We showed that a strong decrease in the uS10 mRNA content may cause a significant deficiency of 40S subunits, resulting in a reduction in the level of 80S monosomes with a noticeable diminution in the efficiency of total translation displayed in the decreased level of polysomes. In general, this state of cells could be characterized as ribosomal shortage. Applying RNA-seq to total and polysome-associated mRNA, we identified genes that were differentially expressed at the levels of the transcriptome and translatome between uS10 knockdown cells and control cells and found that regulation of gene expression in cells with uS10-knockdown occurs at both levels. Therefore, uS10 deficiency caused changes in the transcription of genes that regulate the transcription of other genes. For all of that, changes in the transcription of certain genes, for example, many ribosomal protein genes, were not accompanied by similar changes in the translation of their mRNAs. By analysing up- and downregulated (t)DEGs and (p)DEGs, we found that many genes lack coordination in expression regulation between the transcription and translation levels. We showed that changes in the efficiency of the translation of genes under ribosomal shortage conditions depend on the abundance of the respective mRNAs and the GC content of their CDS.

To measure TE, the ribosome profiling method (Ribo-seq) is most commonly used [19]. This method makes it possible to determine the positions of ribosomal footprints on mRNA and their number, and the term TE in this method actually means the density of ribosomes on mRNA of a particular gene. In the case of the polysomal profiling method (Poly-seq) used in this study, TE is measured by the total amount of mRNA of a particular gene present in polysomes. In this case, the meaning of the term TE can be defined as the frequency of formation of the 80S initiation complex on the mRNA of a particular gene, and perhaps it would be more correct to interpret it as the efficiency of translation initiation. However, it can be seen that there is a direct correlation between the TE in Ribo-seq and the TE in Poly-seq, since the mRNA that enters the translation process at the initiation stage remains translated in polysomes at the elongation stage and the number of ribosomes per mRNA is proportional to the number of initiation events. At the same time, the TE determined in Ribo-seq also depends on the rate of the translation elongation step on a particular mRNA, which is different for different mRNAs, whereas this factor does not affect the TE determined in Poly-seq. In addition, DEGs determined by Ribo-seq and Poly-seq methods are more convenient to compare since they use the same sequencing protocol, which means less error, unlike RNA-seq and Ribo-seq data obtained by different protocols. Therefore, we exploited the Poly-seq method to determine the TEs of genes in this study.

Our data concerning the direction of translatome changes in HEK293T cells with ribosome shortage are generally consistent with and complement the data of other recent studies, in which the analysis of transcriptomes and translatomes under conditions of deficiency of a ribosomal protein was carried out by different methods and on different cell types. So, Khajuria *et al.* [20], performing ribosome profiling in hematopoietic stem and progenitor cells that undergo erythroid lineage commitment, examined changes in global translation and found that the *RPL5* and *RPS19* haploinsufficiency-sensitive transcripts were on average shorter in overall length, while translation of transcripts with longer CDSs increased. In another paper [21], using a microarray assay to determine changes in the transcriptome and translatome of *RPS14*-deficient UT-7/EPO cells, the authors showed that transcript length and codon bias are the key determinants of translation, and short transcripts demonstrate reduced translation efficiency. This suggests that the relationship between the intrinsic characteristics of mRNA and the efficiency of its translation under conditions of ribosomal deficiency is universal.

An important point that should be specifically noted in our results is the weak overlap between the (t)DEG and (p)DEG sets, both up- and downregulated. It would seem that an increase in the transcription of a certain gene should be accompanied by a similar increase in the translation of its mRNA. However, it turned out that this is far from the case. One of the reasons for this phenomenon could be translation buffering [22,23], which may maintain overall cellular levels of some mRNAs even though their translation levels are reduced due to ribosome deficiency. The analysis of genes with altered TE enabled the identification of numerous genes, the change in the translational efficiency of which does not correlate statistically significantly with the change in the overall level of their mRNAs. In some cases, even the opposite trend was observed, as it was, for example, with mRNAs of many ribosomal proteins, when a general decrease in their level was accompanied by an increase in their translation. On the one hand, the decrease in the mRNA level of ribosomal proteins can be explained by a decrease in the activity of *MYC*, a master regulator of ribosome biogenesis, observed in uS10-deficient cells. It is known that the activation of *MYC* in cells activates the transcription of ribosomal genes (for a review, see, e.g. [16]). Therefore, it could be that a decrease in the level of Myc leads to a reduction in the activity of these genes. However, the increase in translational activity of ribosomal genes could not be explained in terms of transcription. Thus, we turned our attention to the intrinsic characteristics of mRNAs and established an opposite correlation between the up- and downregulated (p)DEGs in terms of the cellular abundance and CDS length and GC content of the respective mRNAs. It became clear that the selection of mRNAs towards preferential translation of those that are more abundant and have longer CDSs can occur combinatorically during the ribosomal shortage. In this case, less abundant mRNAs with short CDSs often remain unbound to ribosomes and therefore are not involved in translation.

Global distortion in the pattern of translated mRNAs caused by ribosomal shortage should definitely impair cell viability and lead to multiple disturbances in the functioning of systems of organisms. Obviously, it is rather difficult to correct this situation, since the imbalance between ribosomes and mRNAs could be overcome only through a decrease in the overall level of the transcriptome or through the restoration of the normal level of ribosomes, which is impossible with a lack of an obligatory ribosomal protein. It is known that the synthesis of ribosomal proteins in a cell is usually excessive [24,25]; therefore, under normal conditions, the cell has no ribosome deficiency. If we consider actively proliferating cells, in which the level of translation is extremely high, then the pattern of gene expression will be distorted under conditions of ribosomal shortage. In humans, when one of the alleles of a ribosomal protein-encoding gene is damaged, haploinsufficiency of this protein occurs, which is often the cause of Diamond-Blackfan anemia and some cancer diseases (for review, see e.g. [26–29]). It was already mentioned in the Introduction that uS10 haploinsufficiency is associated with CRC [9]. Somatic mutations in the genes of several ribosomal proteins (*RPL5, RPL10, RPL11, RPS15, RPL22* and others) are associated with tumorigenesis [29]. It appears that actively proliferating intestinal epithelium and erythroid cells may undergo malignant transformation under a deficiency of a ribosomal protein. If our reasoning is correct, then disruption of gene expression at the level of translation caused by the ribosomal shortage in such cells can contribute to their malignant transformation. This assumption is also supported by the results of this study that revealed an increase in the expression of some oncogenes (*TP53, MDM2, JUN, GADD45A*, etc.) in uS10-deficient cells, but this needs to be verified in further works in this field.

## 4. Conclusion

Deficiency of the ribosomal protein uS10 mRNA causes ribosomal shortage and changes in the cellular transcriptome and translatome. Genes that upregulated in the translatome are typically characterized by more abundant mRNAs with longer and GC-enriched sequences than downregulated genes. Changes in the efficiency of the translation of mRNA under conditions of ribosomal shortage can be described by a model of combinatorial selection of mRNAs.

## 5. Methods

### 5.1. Cell culture and transfection

HEK293T (human embryonic kidney) cells (CVCL_0063) were cultivated on 10 cm Petri dishes in Dulbecco's modified Eagle's medium supplemented with 10% fetal calf serum, 100 U ml^−1^ penicillin and 100 µg ml^−1^ streptomycin in a CO_2_ incubator (5% CO_2_) at 37°C. Cells were routinely ensured to be negative for mycoplasma contamination using PCR and mycoplasma-specific primers. Oligoribonucleotides used as uS10 mRNA-specific siRNAs and non-targeting control siRNA (electronic supplementary material, table S1) were prepared as described [30]. Cells were transfected with specific or control siRNAs in four biological replicates at 30% confluence using Lipofectamine 3000 (Invitrogen) according to the manufacturer's protocol and grown 36 h prior to harvest.

### 5.2. Preparation of cell extracts and polysome profiling in sucrose gradients

For harvesting, cells were placed on ice, washed with ice-cold PBS, kept for 10 min on ice with PBS and 0.1 mg ml^−1^ cycloheximide, resuspended, divided into two unequal portions corresponding to the one-fifth and four-fifths parts of cells, and each was pelleted by centrifugation at 500*g* (30 s, 4°C) in a separate Eppendorf tube. One-fifth of the sample (approx. 1.5 million cells) was mixed with TRIzol Reagent (Ambion) to isolate total cellular RNA according to the manufacturer's protocol. Four-fifths of the cells were lysed in 800 µl of ice-cold 20 mM Tris–HCl (pH 7.5) buffer containing 200 mM KCl, 15 mM MgCl_2_, 100 µg ml^−1^ cycloheximide and 1% Triton X-100. The lysate was clarified by a short centrifugation at 13 000 × g for 2 min at 4°C, and the supernatant was layered on a sucrose density gradient (5–50%) in 50 mM Tris–HCl (pH 7.5) buffer containing 100 mM KCl and 12 mM MgCl_2_ pre-formed in Beckman SW40 rotor tubes. Polysome profiles were generated by the ultracentrifugation of samples in a SW40 rotor at 18 500 r.p.m. for 17 h at 4°C and fractioned through the flow cell of a microspectrophotometer with monitoring of the UV absorption profile at A260 nm and collecting fractions on ice. The ribosomal material of fractions corresponding to polysomes and 80S monosomes was precipitated with 0.7 volumes of ice-cold ethanol with the addition of MgCl_2_ up to a 20 mM concentration and pelleted down by centrifugation at 14 000 × *g* for 30 min at 4°C. The pellets were resuspended in water, and RNA associated with polysomes was isolated using TRIzol Reagent as described above.

### 5.3. DNA library preparation and RNA-Seq

RNA extracted from total cell samples and sucrose gradient fractions with TRIzol (Invitrogen) was subsequently isolated by a Purelink RNA Micro Scale Kit (Invitrogen) and DNase treated (DNASE70, Sigma). The RNA integrity index (RIN) was assessed in a Bioanalyzer 2100 (Agilent Technologies) using the RNA 6000 Pico Kit, and RNA quantification was carried out using NanoDrop 1000 (Thermo Scientific) and Qubit (Invitrogen). For 1 µg of each isolated RNA sample, rRNA depletion was performed by RiboCop rRNA Depletion Kits for Human/Mouse/Rat V.2 (Lexogen). DNA libraries were prepared using the MGIEasy RNA Directional Library Prep Set (MGI Tech, China) according to the manufacturer's instructions and subjected to next-generation sequencing (NGS) on the MGIseq-2000 platform, using the 2 × 100 PE sequencing mode (FCL PE100, MGI Tech). All relevant procedures were performed in the SB RAS Genomics Core Facility (ICBFM SB RAS, Novosibirsk, Russia).

### 5.4. Bioinformatics analysis of NGS data

Raw data were assessed for quality with the FastQC (v. 0.11.9) (www.bioinformatics.babraham.ac.uk/projects/fastqc/) (accessed on 9 February 2021) tool and subjected to quality filtering (Trimmomatic 0.39) and adapter trimming (cutadapt) using sequences provided by the manufacturer. The filtered reads were also evaluated for quality and mapped with STAR (v. 2.7.3) using the hg38 reference human genome and Ensembl annotation (release 104). The resulting BAM files were indexed, and their quality was checked using QualimapTool (v. 2.2). Fastq files were deposited in GenBank under the study accession PRJNA976116.

Downstream data analysis was performed as described in [15] with minor modifications. Briefly, the counts table was generated with the application of the Rsubread package (v. 2.10.5) using the featureCounts function with the GTF file (ensembl v. 104) as an annotation in the reverse stranded mode. The biomaRt package (v. 2.52.0) was applied for the annotation. The differential expression analysis was performed with DESeq2 (v. 1.36.0) for RNA-seq and Poly-seq modes separately. The apeglm algorithm was exploited to shrink the LFC values. The genes were treated as differentially expressed ((t)DEGs or (p)DEGs) if *p* adj < 0.05 and the absolute LFC > 0.585 (i.e. only those changes in gene expression levels were taken into account, which were different by more than 50% of the levels in control cells). Genes with altered TE were identified by exploiting DESeq2, as described in the systemPipeR package vignette. Volcano plots were prepared with EnhancedVolcano (v. 1.14.0), and genes passing |LFC| > 0.585 and *p* adj < 0.05 cutoffs are shown as coloured dots. The scatter plot for genes with altered TE was generated with ggplot2 using ratios (KD/NC) of log2-transformed mean normalized read values (plus 1 pseudocount) for RNA-seq and Poly-seq modes. Genes with significant change of TE (|LFC| > 0.322, *p* adj < 0.05) are colour-coded. TPM (transcripts per million) counts were estimated with the kallisto (v. 0.46.2) quant function using Homo_sapiens.GRCh38.cdna.all.fa as a transcript reference. Codon frequencies for each CDS were extracted with coRdon (v. 1.14.0). The effect size of differences in codon frequencies between up- and downregulated (p)DEGs sets as well as neutral (p)DEGs (TPM_RS_NC > 0) were estimated as log2-transformed ratios of mean codon frequencies (mean for a certain set of CDS), and the significance of changes was tested by *t*-test (codon frequency values for each CDS were used); the obtained *p*-values were corrected using the Benjamini–Hochberg approach using the R stats p.adjust function. GO enrichment analysis was performed using the GeneOntology web service (release date 5 October 2023; reference list: *Homo sapiens*; test type: FISHER; correction: FDR). Visualization of the analysis was performed in RStudio as described in [15] and in SCImago Graphica. GraphPad Prism v9 (GraphPad, USA) and OriginPro (Origin lab Corp., USA) were used for statistical analyses.

### 5.5. RT-qPCR

Reverse transcription (RT) was carried out using 2.5 µg of RNA from the aliquots of the respective samples of isolated total or polysome-associated mRNA, 100 pmol of a random hexamer primer and 20 U of MMLV reverse transcriptase according to Gopanenko *et al.* [31]. The resulting cDNA was then used for qPCR analysis, which was performed using SYTO9 fluorescent dye (Thermo Fischer Scientific), hot-start HS-Taq polymerase (Biolabmix) and appropriate gene-specific primers (electronic supplementary material, table S2). The experiments were performed in four biological replicates. Relative levels of gene expression were quantified using integrated LightCycler 96 (Roche, Basel, Switzerland) software using the *GAPDH* gene expression level as a reference.

## Data Availability

RNA-seq data were submitted to GenBank under the study accession number PRJNA976116. Fastq files were deposited in GenBank under the study accession number PRJNA976116. Supplementary material is available online [32].
